# Exploring the Mediating Role of Social Participation in Reducing Caregiver Burdens in Dementia Care

**DOI:** 10.1002/alz.095404

**Published:** 2025-01-09

**Authors:** Suyeong Bae, Ickpyo Hong

**Affiliations:** ^1^ Yonsei University, Wonju Korea, Republic of (South); ^2^ Yonsei University, Wonju, Gangwon‐do Korea, Republic of (South)

## Abstract

**Background:**

The increasing global incidence of dementia is accompanied by rising burdens for caregivers, who face physical and emotional challenges such as depression and pain. Existing research primarily explores the negative impacts of caregiving on quality of life, health, and finances, alongside the benefits of social support and specific programs. However, such approaches might be relatively passive. Our study aims to examine the mediation effect of social participation between caregivers and their burdens. We expect the study finding could be used as an active strategy that significantly alleviates caregiver burdens.

**Method:**

We used the data of caregivers and recipients from the 2022 National Health and Aging Trends Study and National Study of Caregiving IV. The dependent variables were self‐reported questionnaires that assess physical and emotional burdens. Social participation was used as a mediator, and the independent variable was whether the caregiver cared for recipients with dementia or not. Structural equation modeling was utilized to investigate the mediating role of social participation in the relationship between caregivers and the physical and emotional burdens they experience.

**Result:**

The final sample was 1,836 caregivers, and they consisted of 595 males (32.41%) and 1241 females (67.59%). The model fit value indicated that the model fits the data (chi‐square value = 322.795, df = 60, *p*‐value = 0.000; RMSEA = 0.049; CFI = 0.938; TLI = 0.919; SRMR = 0.043). The caregivers who care for recipients with dementia were negatively associated with social participation (standardized coefficient = −0.114, *p*‐value <.0001). Social participation was negatively associated with the physical and emotional burden (standardized coefficient = −0.230, *p*‐value <.0001; standardized coefficient = −0.208, *p*‐value <.0001, respectively). There were no significant direct paths between caregivers who care for recipients with dementia and physical burden, emotional burden each other.

**Conclusion:**

The study findings can help recognize the role of social participation in the caregivers who care for recipients with dementia by understanding the positive effect of social participation. In addition, the findings suggest that social participation can reduce the physical and emotional burden of caregivers who care for recipients with dementia.

